# Combustion Synthesis of Aluminum Oxynitride in Loose Powder Beds

**DOI:** 10.3390/ma14154182

**Published:** 2021-07-27

**Authors:** Alan Wilmański, Magdalena Zarzecka-Napierała, Zbigniew Pędzich

**Affiliations:** Faculty of Materials Science and Ceramics, AGH University of Science and Technology, 30 Mickiewicz Av., 30-059 Kraków, Poland; zarzecka@agh.edu.pl

**Keywords:** aluminum oxynitride, combustion synthesis, salt-assisted, SHS, AlON

## Abstract

This paper describes combusting loose powder beds of mixtures of aluminum metal powders and aluminum oxide powders with various grain sizes under various nitrogen pressure. The synthesis conditions required at least 20/80 weight ratio of aluminum metal powder to alumina powder in the mix to reach approximately 80 wt% of γ-AlON in the products. Finely ground fused white alumina with a mean grain size of 5 μm was sufficient to achieve results similar to very fine alumina with 0.3 μm grains. A lower nitrogen pressure of 1 MPa provided good results, allowing a less robust apparatus to be used. The salt-assisted combustion synthesis upon addition of 10 wt% of ammonium nitrite resulted in a slight increase in product yield and allowed lower aluminum metal powder content in mixes to be ignited. Increasing the charge mass five times resulted in a very similar γ-AlON yield, providing a promising technology for scaling up. Synthesis in loose powder beds could be utilized for effective production of relatively cheap and uniform AlON powder, which could be easily prepared for forming and sintering without intensive grounding and milling, which usually introduce serious contamination.

## 1. Introduction

The binary Al_2_O_3_–AlN system contains a solid solution known as aluminum oxynitride with a cubic structure usually abbreviated as γ-AlON [1]. The compound was first synthesized in 1959 by Yamaguchi and Yanagida but gained more interest after the first successful sintering of a transparent specimen by McCauley and Corbin [2]. The material can achieve excellent transmittance from ultraviolet to mid-infrared wavelengths [3]. These ceramics have various favorable properties, such as good mechanical strength, temperature stability, and chemical resistance, that allow them to be applied as transparent armor, missile domes, refractory materials, optoelectronics, and high temperature windows [4,5,6].

Manufacturing of aluminum oxynitride ceramics usually starts with the synthesis of γ-AlON powder with one of the well-known methods, i.e., carbothermal reduction and nitridation (CRN) [7,8,9,10,11] or solid-state synthesis (SS) [12,13,14,15], or one less frequently used, such as aluminothermic reduction and nitridation (ARN) [16,17] or self-propagating high temperature synthesis (SHS) [18,19,20,21,22]. The main drawbacks of the most popular processes are high temperatures of over 1650 °C and prolonged soaking times. Dealing with these problems by employing the sol-gel method results in other disadvantages, such as high cost of chemical reactants and long gel preparation times, producing, in return, extremely fine γ-AlON powders after synthesis [23,24]. Another concern of the CRN method relates to oxidizing the remaining carbon, present in the powder batch post reaction, in open air furnaces at 600–700 °C for a few hours that can lead to a change in the aluminum oxynitride stoichiometry, which has not been addressed in any of the publications on CRN. An additional disadvantage is a necessity of a secondary operation before the powders can be used to form green bodies. A recent paper describes the oxidation behavior of coarse (30 μm) γ-AlON powders in air at temperatures above 800 °C [25]. The powders were prepared by the CRN technique and the referred publication, on the basis of which the powders were prepared, does not provide any data on the procedure. What it does provide is a DTA plot ranging from 400 °C and presents a small constant increase in exothermic reaction taking place that cannot be attributed only to residual carbon oxidation, as the fine grains of γ-AlON may also oxidize.

The SHS has the potential of being a cheap and fast alternative of producing less pure γ-AlON. Several papers describe different reaction environments with the key component being a mixture of Al_2_O_3_ and Al. The first attempts were conducted in air at ambient pressure, giving the best results within 40–50 wt% of aluminum [18]. The obtained powders contained a significant number of impurities consisting of Al_2_O_3_, AlN, and melted aluminum. Zientara et al. investigated igniting mixtures of 15–50 wt% Al with Al_2_O_3_ under 2.5 MPa nitrogen [19]. At low aluminum contents (15, 20%), γ-AlON yield was low (c.a. 30%); when increasing the proportion of Al, the synthesis resulted in a higher, i.e., 40–50 wt%, γ phase and a significant decrease of residual Al_2_O_3_, simultaneously having increased amounts of AlN, 21R, and unreacted aluminum. The same year, another paper described mixtures of 30 and 50 wt% Al ignited in nitrogen atmosphere under 1, 3, and 5 MPa pressure, resulting in mixtures of γ-AlON as the main phase and residual AlN, Al_2_O_3_, and Al [20]. Samples with 30% Al synthesized under 3 and 5 MPa N_2_ had no residual aluminum present; the authors do not present number values for phase compositions of the resulting powders. The addition of aluminum nitrite nonahydrate and high nitrogen pressure were examined in [21]. A fixed amount of 40 wt% Al was used, and 10–40 wt% of aluminum nitrite, in exchange for aluminum oxide, was mixed and pressed to 60% relative density. Samples were ignited under 100 MPa nitrogen, forming a porous structure. All samples were composed mostly of γ-AlON with impurities consisting of Al (in low nitrite sample), 21R, and AlN (in high nitrite sample). Another approach with an oxidizer was conducted by Akopdzhanyan et al. [22]. The proportion of Al/Al_2_O_3_ was fixed at 21/79 and 25/75 wt% with up to a 17 wt% addition of Mg(ClO_4_) under 5–100 MPa N_2_ pressure. Perchlorate acted as an oxidizer leaving MgCl_2_ as a minor impurity that was washed by boiling in deionized water. The amount of magnesium that can enter the solid solution in aluminum oxynitride was not taken into account and not determined. The best result was given by the lower aluminum mix with 11.4 wt% perchlorate synthesized under 20 MPa nitrogen pressure followed by washing, presenting no detectable aluminum and magnesium compounds on the diffraction pattern.

None of the cited papers present the influence of the different grain sizes of powder substrates on the γ-AlON formation and yield. There is also a lack of information on the influence of lower nitrogen pressure on the mentioned process. Most of the available literature describes synthesis in powder compacts rather than loose powders. Synthesis in loose powder beds could be utilized for effective production of relatively cheap and uniform AlON powder which could be easily prepared for forming and sintering without intensive grounding and milling, which usually introduce serious contamination. In this work, we present a broad approach to investigate the influence of the grain size of aluminum metal and oxide powders, nitrogen pressure in the reaction chamber, proportion of Al/Al_2_O_3_, and the use of ammonium nitrite in loose powder beds on phase composition and γ-AlON phase yield of SHS-derived powders.

## 2. Materials and Methods

**Figure 1:**
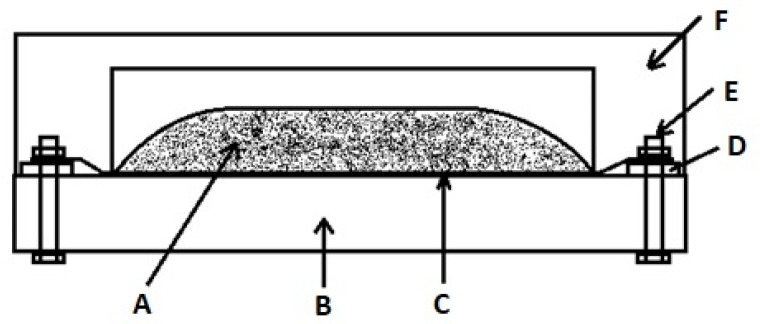
Schematic of the reactor setup. (**A**) powder bed, (**B**) high temperature brick, (**C**) graphite foil boat, (**D**) copper contact electrode, (**E**) mounting screw and nut, (**F**) insulating wool.

Commercial powders included Al_2_O_3_ (KOS Korund, Koło, Poland, EA-320 d_50_ = 55 μm, EA-1200 d_50_ = 5 μm, Almatis CT3000 LS SG d_50_ = 0.3 μm), Al (Sun Chemicals, Skawina, Polnad, Al-7 d_50_ = 8 μm, Al-32 d_50_ = 11 μm, Al-63 d_50_ = 49 μm, Al-150 d_50_ = 200 μm), NH_4_NO_3_ (Avantor, Gliwice, Poland, <250 μm), and N_2_ (Air Liquide, Cracow, Poland). Powder purity was over 99.5%, and nitrogen was 99.99%. Powders were weighed and mixed in a rotating vibration mill using 5 mm alumina balls in isopropanol for 1h. The powders were dried in a laboratory oven at 60 °C. A 100 g powder batch was placed on a boat-shaped graphite foil and surrounded by high temperature insulating wool. Ends of the foil were connected to copper electrodes. A schematic representation of the SHS reactor setup is presented in Figure 1. This setup was placed in a pressure chamber which was purged with nitrogen for a few seconds; then, the release valve was closed, and the chamber was evacuated. The chamber was filled with nitrogen to reach the designed pressure. The powder ignition was done by passing current through the graphite foil for 15 s. After the reactor cooled down (0.5–2 h, depending on nitrogen pressure used) the slightly sintered powders were broken down by hand and crushed in a laboratory jaw crusher.

Phase composition of the powders was analyzed by X-ray diffraction (X’Pert Pro, PANalytical, Malvern, UK), and the Rietveld refinement allowed determining the quantitative phase content. Powder morphology was observed using scanning electron microscopy (FEI Nova Nano SEM 200, Thermo Fisher Scientific, Hillsboro, OR, USA). The time–temperature profile has not been measured as the reaction zone is fully insulated with aluminasilicate wool, and the reaction chamber was not equipped with an outlet for a thermocouple.

## 3. Results

**Figure 2:**
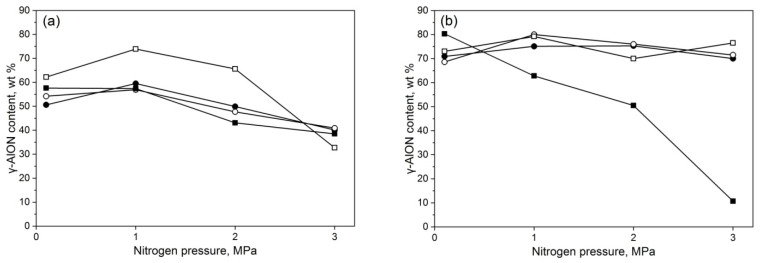

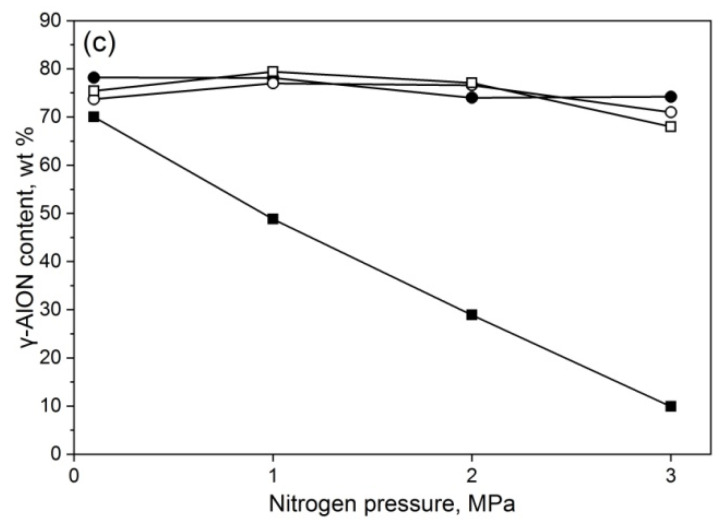
Aluminum oxynitride yield achieved by the means of SHS method from various aluminum metal powders: Al-7 (●), Al-32 (○), Al-63 (□), and Al-150 (■) and under varied nitrogen pressures for different substrates: (**a**) coarse EA-320, (**b**) medium EA-1200, (**c**) fine CT3000 alumina.

The first part of the experiment was designed to determine the optimal grain size of aluminum and aluminum oxide powders and an optimal nitrogen pressure. The weight ratio of Al/Al_2_O_3_ was fixed at 20/80 as this was known to sustain the reaction self-propagation. The nitrogen pressure was established as 0.1, 1, 2, and 3 MPa. The outcome of the synthesis was rated for the amount of γ-AlON, secondary oxynitride, and residual aluminum. The amount of γ-AlON obtained in this part is presented in Figure 2.

The coarse alumina substrate presented the lowest tendency for γ-AlON formation. The highest yield was achieved with Al-63 under 1 MPa nitrogen (74% γ-AlON). What is worth noticing is that no residual aluminum was detected, and the sum of present oxynitrides was calculated to be 87.5%. The medium and fine alumina substrates performed similar to each other. Both achieved between 70 and 80% γ-AlON at every pressure in combination with Al-7, -32, and -63, and the coarse aluminum showed a steep decrease with increasing synthesis pressure. The highest amount of γ-AlON phase was achieved at 1 MPa N_2_ pressure and Al-63, the same as for the coarse Al_2_O_3_ substrate, and additionally with Al-32 combined with medium alumina; however, there was a little residual aluminum detected. The sum of oxynitrides was found to be slightly higher for the medium alumina substrate than for the fine alumina one, reaching 89% and 87.7%, respectively. Both alumina substrates showed a high conversion rate to γ-AlON with coarse aluminum powder under low pressure and a sharp decline with increasing pressure. These results may prove that using ultra fine alumina and aluminum metal powders does not give any benefit in return over cheaper and coarser resources when reacted in loose beds. The same applies for the nitrogen pressure in the reaction chamber. Judging from the above results, the powders of Al-63 and EA-1200 and a pressure of 1 MPa of nitrogen gas were chosen for further investigations.

Substrate composition in the ratio of 20/80 for Al/Al_2_O_3_ reacting with nitrogen resulted in a 50/50 mol ratio of AlN/Al_2_O_3_, which is richer in nitrogen than any aluminum oxynitride in the phase diagrams presented in the literature. The next step was to determine if lowering the aluminum ratio could be a viable solution for γ-AlON yield increase. For this, the aluminum/alumina ratio was decremented in 1% steps until reaching 14/86, which did not ignite.

**Figure 3:**
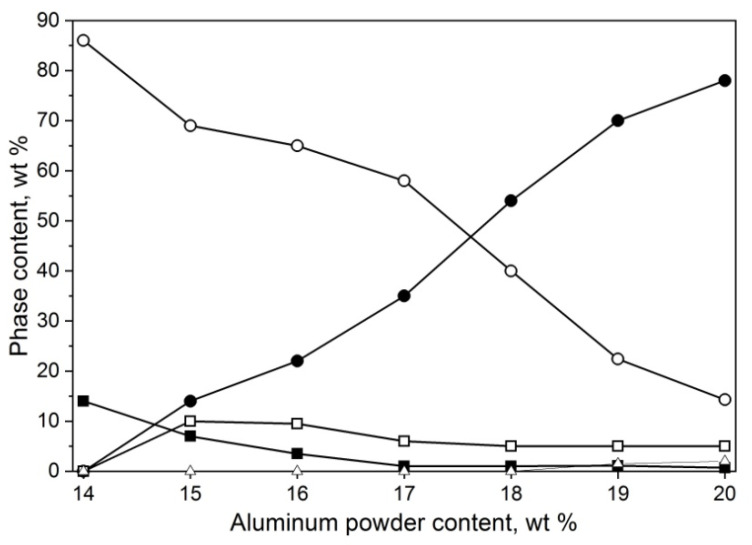
Phase composition of powder mixtures obtained by the means of SHS method from mixes of powders with 14–20 wt% aluminum content and medium alumina EA-1200 powder synthesized under 1 MPa nitrogen, γ-AlON (●), Al_2_O_3_ (○), AlN (□), Al (■), and Al_7_O_3_N_5_ (∆).

The resulting powder compositions are presented in Figure 3. The powder mixture with the lowest amount of aluminum did not ignite. The 15 and 16% batches were able to ignite, but the reaction was unsustainable, with much of the aluminum left unreacted, partially nitrided to AlN and partially being able to react with alumina to form 13 and 22% γ-AlON, respectively. From 17% Al, there is a noticeable linear increase in the γ-AlON phase yield and consumption of alumina; the residual AlN and Al stabilize at around 5 and 0.5%, respectively. In the batch containing 19% Al, the 21R phase appears. This experiment showed no benefit from decreasing the amount of aluminum in the starting powder by even one percent.

**Figure 4:**
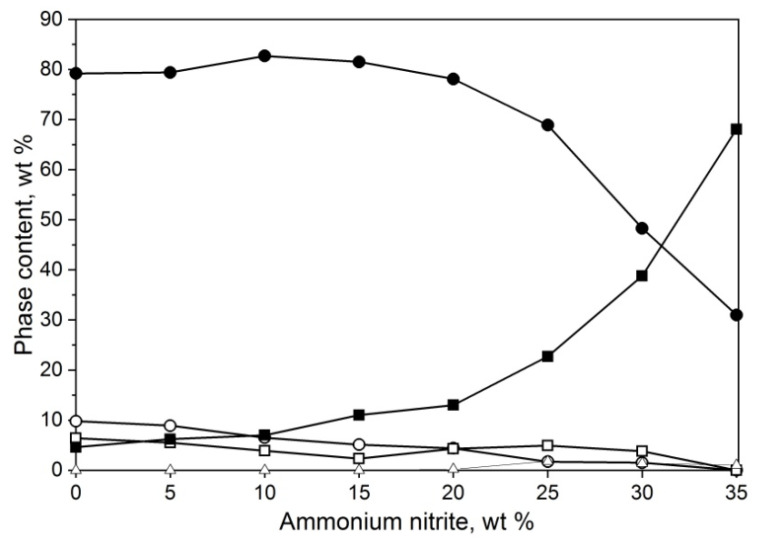
Phase composition of powders obtained by the means of SHS method from mixtures containing different amount of ammonium nitrite, γ-AlON (●), Al_2_O_3_ (○), AlN (□), Al (■), and Al_7_O_3_N_5_ (∆).

The third part of the experiment was to investigate the influence of ammonium nitrite on the system. The amount of salt used was 5–35 wt%, keeping the Al/Al_2_O_3_ ratio fixed at 20/80. Phase compositions of the resulting powders are presented in Figure 4. A slight increase in γ-AlON synthesis occurred for samples containing 10 and 15% nitrite. Adding more salt, above 20%, resulted in a sharp decrease in oxynitride and a rise in alumina content. The powder bed after the reaction with 20% or more salt was visibly melted with cavities in the middle, with graphite foil stuck to the bottom, and being hard to break by hand.

**Figure 5:**
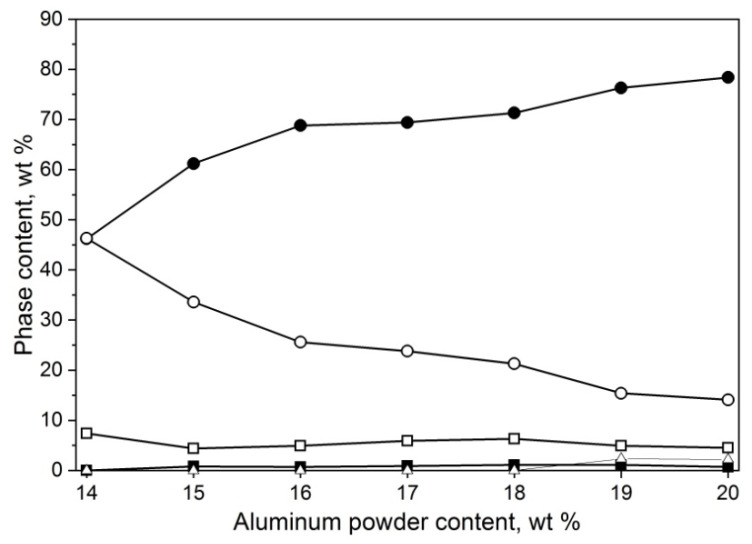
Phase composition of powder mixtures obtained by the means of SHS method from mixes of powders with 14–20 wt% aluminum content with medium alumina under 1 MPa nitrogen with 10% ammonium nitrite, γ-AlON (●), Al_2_O_3_ (○), AlN (□), Al (■), and Al_7_O_3_N_5_ (∆).

The experiment with the reduced amount of aluminum was repeated using 10% ammonium nitrite to determine if the salt addition could help with the synthesis. Figure 5 shows the phase composition of produced powders. The most noteworthy fact is that the mix of the lowest aluminum content not only ignited but also allowed for the reaction zone to go through the whole powder bed, yielding around 46% γ-AlON and the same amount of Al_2_O_3_. More aluminum in the mixtures resulted in increasing the amount of aluminum oxynitride yield and decreasing the alumina content in the system; however, AlN was found to be retained at around 5 to 8 wt% without any aluminum detected.

**Figure 6:**
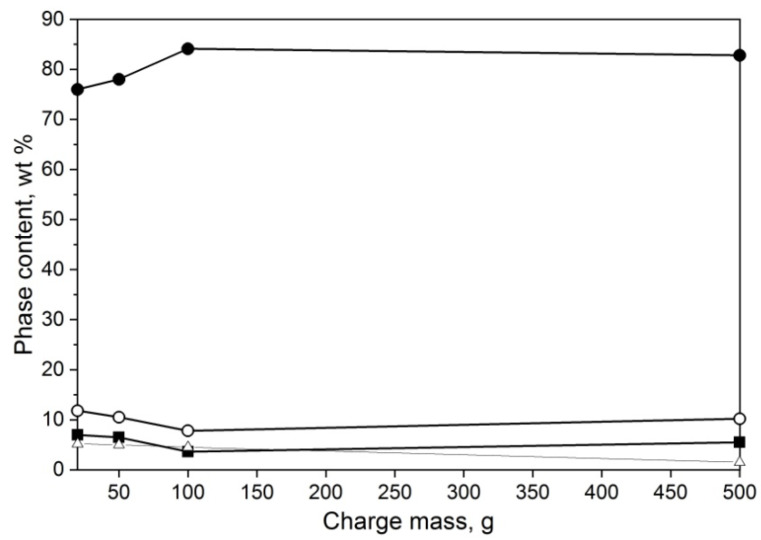
Phase composition of powder mixtures obtained by the means of SHS method for different powder mass batches, γ-AlON (●), Al_2_O_3_ (○), AlN (■), and Al_7_O_3_N_5_ (∆).

Finally, the scalability of the setup was investigated. Mixes of 20/80 aluminum ratio with 10% ammonium nitrate were prepared and weighed in batches of 20, 50, 100, and 500 g to be ignited in 1 MPa nitrogen. The smaller batches were less successful with γ-AlON synthesis; however, the 500 g charge presented less than a 2% decrease in γ-AlON formation. The results are presented in Figure 6.

**Figure 7:**
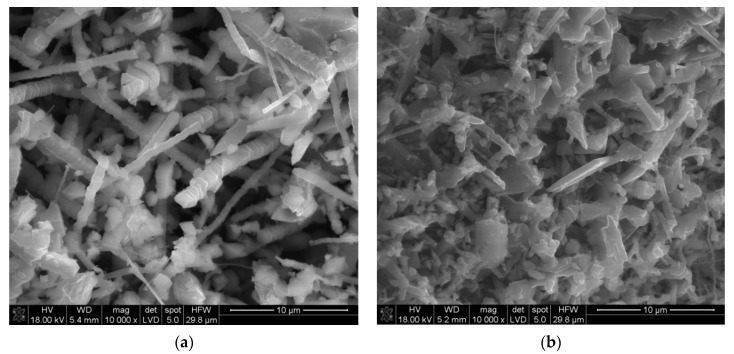
SEM images of powders after SHS, (**a**) medium alumina with Al-63 under 1 MPa, (**b**) coarse alumina with Al-63 under 1 MPa.

The morphology of the achieved powders is visualized in Figure 7. It presents elongated columnar shapes and whiskers with hexagonal cross sections and layered structures. Flat platelets can be also seen in the images.

## 4. Discussion

As expected, the least amount of γ-AlON phase was synthesized with the coarse alumina substrate due to the longest distance that the diffusing atoms have to travel. Additionally, a larger grain poses a higher resistance for reaction heat to raise the temperature inside the grain. The similarity in the synthesis outcome for alumina powders with a mean grain size of 5 and 0.3 μm means the threshold for the oxide substrate is at least 5 μm. The similarities continue with the proposed optimal reaction pressure as well as the aluminum grain size. Contrary to cited articles, pressures lower than 5 MPa were sufficient for nitridation of whole aluminum in the powder batch. Such conditions allow most of the formed aluminum nitride to react with the surrounding alumina.

The basic reaction allowing for the reaction to be self-sustaining is

2Al + N_2_ → 2AlN + Q (1)

The heat (Q) released in first reaction heats up adjacent grains and triggers the aluminum ones to also react with nitrogen. Aluminum is always covered with a passivation oxide layer that prevents it from further reaction; however, during the initial heating, the grain expands more than the oxide, exposing a clean surface for the nitrogen to react. The amount of heat generated melts the grain and causes partial or complete evaporation. Aluminum vapors are very reactive and bond with nitrogen immediately. The freshly formed AlN condenses on surrounding Al_2_O_3_ grains that had already been heated and starts the secondary reaction:

xAlN + yAl_2_O_3_ → Al_x+2y_O_3y_N_x_ (2)

From the appearance of the reacted powder bed, the reaction did not exceed 2050 °C, which is the melting temperature of aluminum oxide, as no sign of macroscopic melting could be found. Only parts of the powder near the graphite foil ends that ignite the reaction are more sintered, being heated from the reaction and resistive heating simultaneously. Nevertheless, at temperatures below melting, alumina becomes volatile and may react with AlN in gaseous phase. The SEM images in Figure 7 depict elongated columnar shapes with hexagonal cross sections and layered growth structures and support both mechanisms of V-S and V-L-S as proposed by Zientara et al. [19]. These structures can be composed of AlN and γ-AlON as EDS shows the presence of oxygen and nitrogen in varying amounts, but this method is not precise enough to determine the stoichiometry of the examined spot. Both compounds can form such structures, AlN growing in the [0001] direction and the γ phase in the [111] direction, forming hexagonal crystal forms. There are also more solid grainy structures that suggest a solid-state synthesis with interdiffusion taking place between nitride and oxide. As our apparatus is not equipped with a thermocouple or pyrometer and also does not have a window, we can only estimate the time of the parts of the powder bed remaining at temperatures above 1650 °C which promotes γ-AlON synthesis. In general, the reactor cooled down slower during the low-pressure experiments.

According to the literature data on aluminum nitriding in the SHS regime at a lower pressure range, an increase in pressure speeds up the reaction front propagation [26]. For γ-AlON synthesis, it was observed that at high pressures above 50 MPa, a further increase of pressure slows down the reaction [23]. The reference also presents a data point at 20 MPa with a reaction front having half the velocity of 50 MPa. What is more, the experiment was conducted on pressed pellets, and the current is run on loose powder beds; consequently, the velocities may differ significantly. The incomplete conversion of substrates may be also attributed to less particle contacts and a greater distance from each other. These conditions hinder the formation of oxynitride, at the same time allowing easier nitrogen penetration into to the bottom of the powder bed. What also has to be taken into consideration is the edge effect; even though the whole batch could be removed from the reactor intact, the conditions prevailing in layers in contact with graphite and the top layer may contribute to an overall lower yield.

The use of ammonium nitrite fulfills a number of functions. The salt melts before it decomposes, thus creating capillary action and pulling neighboring grains closer. The decomposition creates active nitrous oxide, oxygen, and nitrogen, aiding in nitriding and oxidizing aluminum, increasing temperature and flame front velocity. Another advantage of using an ammonium salt is the absence of any additional metal cations dissolved in the oxynitride lattice or metal salts that have to be further removed by washing and could still remain in the product. The ammonium nitrite addition has a positive effect on the synthesis yield of up to 10 wt%; a further increase in salt content led to more aluminum being oxidized than nitrided due to the higher affinity of aluminum to oxygen than to nitrogen. The temperature increase above the γ-alon melting temperature of 2150 °C can be inferred from strongly sintered and partially melted products with less and less of the desired oxynitride.

Experiments on scalability are promising with batches five times larger (500 g) than the standard ones (100 g) used in the presented investigation. The amount produced in the scale up experiment only required a redesign of the graphite boat to the largest size that could fit the current reactor construction. The enlarged powder bed post reaction was very similar to the standard one, and the γ-AlON yield was also very similar. This experiment gives promising insight into the possibility of scaling up to several kilograms of product in one charge.

The product obtained from a 20/80 mixture results in an Al_2_O_3_:AlN ratio equal to 1:1, which is twice the amount of nitride required to form the γ-AlON phase. This fact can be addressed during preparation for sintering by mixing it with an appropriate amount of alumina to compensate the excess nitrogen in the product. This issue is going to be presented and discussed in further publications.

## 5. Conclusions

The synthesis of γ aluminum oxynitride using the SHS technique on a loose powder bed has been studied. The influence of alumina and aluminum particle size as well as nitrogen pressure and ammonium nitrite salt on phase composition and γ-AlON phase yield was presented. The tested procedure allowed us to achieve products with over 80% γ-AlON content, having no residual aluminum where obtained. Promising results could be achieved using relatively cheap resources. The substrates used for the experiments have particle sizes larger than those presented in the literature as of yet. The ignition of homogenously mixed alumina powders with a mean grain size of 5 μm or smaller, with aluminum powder with a mean grain size of 50 μm or smaller, and with the addition of 10 wt% of ammonium nitrite in 1 MPa pressure nitrogen results in over 80% γ-AlON yield. Presynthesizing γ-AlON using SHS and cheap raw materials followed by a lower nitrogen pressure than that reported in the literature could be a promising route to scaling up the technology and could be beneficial for transparent armor manufacturers. Additionally, low pressures around 1 MPa require a less robust reactor apparatus, which is both cheaper and safer.

The conducted experiments were the first reported in the literature to attempt using ammonium nitrite as an aid in γ-AlON synthesis by the means of the SHS method.

Increasing the test batch five times resulted in a consistent and very similar outcome as that achieved during the standard tests. Flame front velocity, temperatures reached, cooling rate, and edge conditions are still parameters which should be investigated for process optimization.

## Data Availability

The data presented in this study are available on request from the corresponding author.
